# Essex County Hospital

**Published:** 1910-12-10

**Authors:** B. H. Nicholson, Sydney Curl, Edward Chichester, Leigh Day, Wm. F. A. Clowes, Philip Laver

**Affiliations:** Chairman; Hon. Secretary


### Editor's Letter-Box.

### ESSEX COUNTY HOSPITAL.

*To the Editor of* The Hospital.

Dear Sir,—In an article on the Essex County Hospital, written by Sir Henry Burdett and published in your issue of November 12, there is a paragraph headed "Administration and Management." This paragraph criticises the institution in a somewhat adverse manner. Though deeply grateful to Sir Henry Burdett for his great assistance to the Essex County Hospital and for the trouble he has taken —and taken at very great personal inconvenience, as we know—to assist the committee in its efforts to put the hospital finances on a better footing, we cannot help feeling great regret at the strictures he has passed on the nursing department—strictures which, we believe, he never would have passed if time had allowed him to go thoroughly into the matter and if he had seen the hospital at work daily as we do.

Of course, it is extremely difficult to controvert such general criticism as "there is an absence of organised control, efficient supervision, and discipline within the hospital"; but we are bound to say that in our opinion the discipline of the nursing staff and the orderly arrangement and conduct of the work is decidedly good. There can be no two opinions among those conversant with the facts as to the vast improvement in the internal working of the hospital within the last few years, and it must be a very great source of worry and discouragement to the matron, who has worked so hard; and we cannot but wonder how Sir Henry arrived at such an unfavourable conclusion.

The more specific points raised in the paragraph in question are more easily dealt with: the medical staff can assure Sir Henry that they have never found the wards or operating-theatre unready at any time; dust in the operating-theatre is a thing with which the surgical staff is unacquainted. It is true that there are ten flock-pillows in the institution; but these no doubt will, now that attention has been drawn to them, be done away with. The tuberculous patients who sleep out of doors have always been specially asked whether they wished for more blankets, and have always been allowed as many as they like to have. It is not the case that one sister and one nurse have charge of twenty-three beds. Each ward has one sister, one nurse, and one probationer. One ward only has twenty-three beds, and there extra help is provided when the work is heavy. All the other wards have fewer beds with one exception, the female medical ward, where there are eighteen beds and six cots. Here, however, an extra nurse is employed.—We are, yours truly,

B. H. Nicholson, M.D., etc., Edin. (Chairman).
Sydney Curl, M.D. Camb., M.R.C.P. Lond.
Edward Chichester, M.B. Lond., M.R.C.S., etc.
Leigh Day, M.D. Oxon.
Wm. F. A. Clowes, M.R.C.S., etc.
Philip Laver, M.R.C.S., etc. (Hon. Secretary).

[We gladly publish the above letter. If our correspondents will read Sir Henry Burdett's report again they will find that he passed no strictures on the nursing department, which is not even mentioned. Sir Henry describes the atmosphere of the hospital as he found it at the time of his inspection, and in the result expresses his opinion that "steps should be taken to amend the present system so as to make the discipline and orderly arrangement of the hospital more perfect than it is at present." Sir Henry's standard of comparison is that of a modern up-to-date hospital. We have no doubt that our correspondents are cordially in agreement with the Committee that it is desirable to work for the attainment of this high standard at Colchester.—Ed.]

